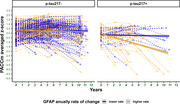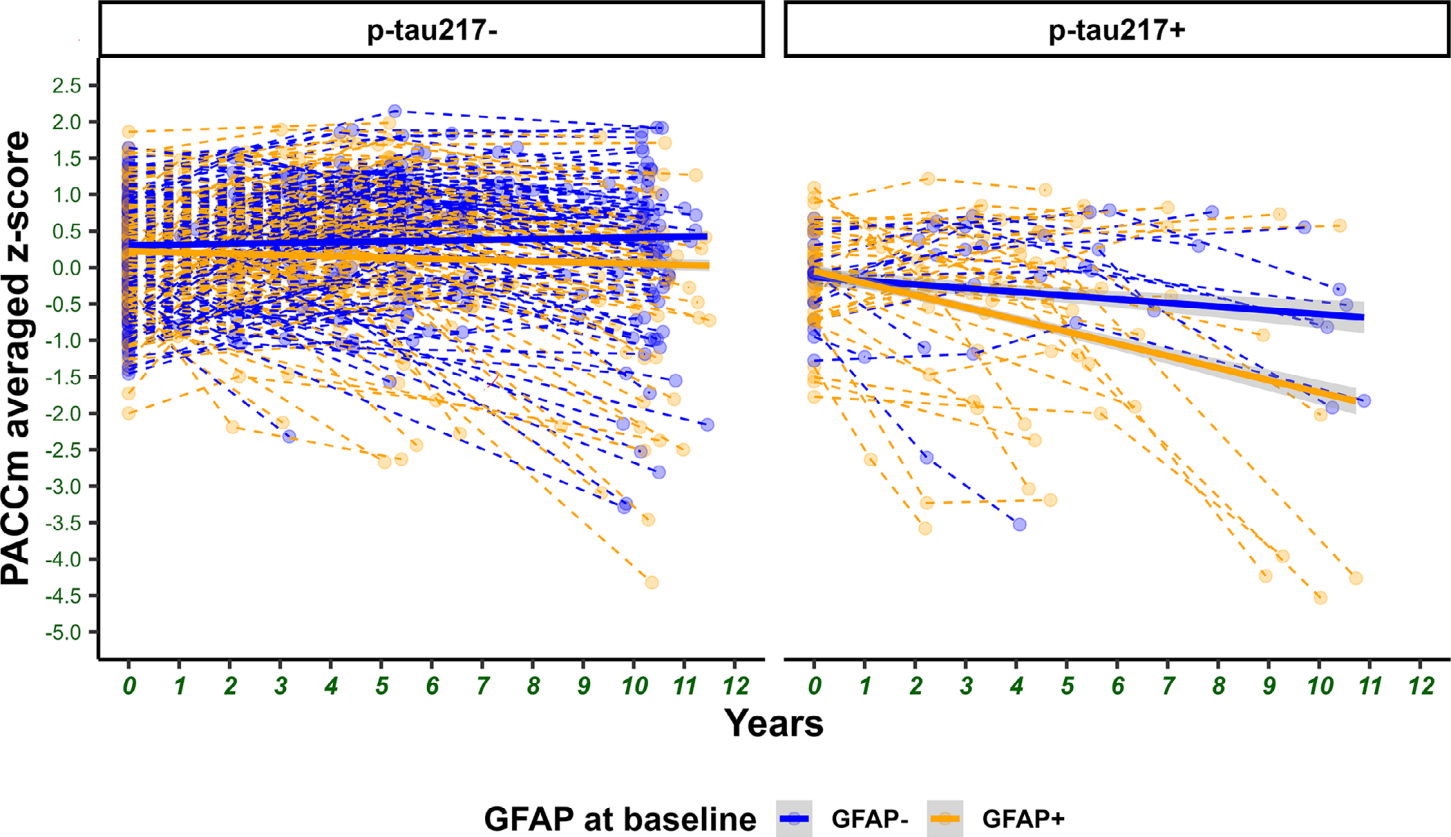# Modulatory effect of glial fibrillary acidic protein (GFAP) levels on the association between plasma *p*‐tau217 levels and cognitive decline in older adults

**DOI:** 10.1002/alz70856_106267

**Published:** 2026-01-08

**Authors:** Elizabeth Lucia Valeriano‐Lorenzo, Mario Ricciardi, Sonia Wagner, Minerva Martinez, Belén Frades, María Ascensión Zea‐Sevilla, Meritxell Valentí, Teodoro del Ser, Pascual Sánchez‐Juan

**Affiliations:** ^1^ Alzheimer's Centre Reina Sofia Foundation‐CIEN Foundation‐ISCIII, Madrid, Madrid, Spain; ^2^ Alzheimer's Center Reina Sofia‐CIEN Foundation, Madrid, Madrid, Spain; ^3^ CIEN Foundation, Reina Sofia Alzheimer Center, ISCIII, Madrid, Madrid, Spain

## Abstract

**Background:**

Recent studies demonstrate an association between glial fibrillary acidic protein (GFAP) levels and post‐mortem tau pathology and highlighted its role as a link between amyloid and tau pathology in Alzheimer's disease (AD) [Sánchez‐Juan, Pascual, et al., Brain 147.5 (2024): 1667‐1679.]. Additionally, growing evidence suggests that neuroinflammation may modulate the effects of tau spread on cognitive impairment in AD [Peretti, Débora E., et al., Brain 147.12 (2024): 4094‐4104.]. In this context, this study aims to assess the synergistic effect on cognitive decline of plasma levels of GFAP, and *p*‐tau217, as surrogate biomarkers of reactive astrogliosis and AD pathophysiology respectively.

**Method:**

We included 354 cognitively unimpaired individuals (mean age = 74.1±3.6 years; 61.9% women) from the Spanish Vallecas Project, a longitudinal study of elderly volunteers. Plasma GFAP concentrations were measured at three‐time points of the follow‐up (baseline, intermediate, and final visits) using the highly sensitive single molecular array (SIMOA) and those >141.87pg/mL were considered high (GFAP+), additionally, an annual rate of change >10.26pg/mL was considered fast (GFAP_fast.rate_). Plasma *p*‐tau217 levels at baseline were measured using the Lumipulse platform and those >0.247pg/mL were considered high (*p*‐tau217+). Cognition was assessed annually over 10 years using a modified Preclinical Alzheimer Cognitive Composite (PACCm). Linear mixed‐effect models were used to analyze the longitudinal trajectories of cognition and GFAP and to examine the moderation effect of GFAP controlling for education, sex, age and ApoE4.

**Result:**

58(16,4%) participants were classified as *p*‐tau217+, 176 (49,7%) as GFAP+, and 173(48,9%) showed GFAP_fast.rate_. Participants with *p*‐tau217+ and higher annual GFAPfast.rate showed a significantly faster cognitive decline (βTime*pTau217*GFAP_fast.rate_ = ‐0.158; *p* <0.001) than those with non‐GFAP_fast.rate_ (Figure 1). However, baseline levels of GFAP and *p*‐tau217 did not show a significant interaction (βTime*pTau217*GFAP_baseline_ = ‐0.076; *p* = 0.094) (Figure 2).

**Conclusion:**

Our results indicate that GFAP_fast.rate_ modulates the relationship between *p*‐tau217 and cognitive decline independently of age, sex and ApoE4, with a stronger decline observed in individuals with *p*‐tau217+ who likely present a reactive astrogliosis. Further studies are needed to examine the mechanisms underlying the interaction between reactive astrogliosis and tau spread in the AD continuum. This could help us better understand neuroinflammation as a potential target for therapeutic strategies.